# RETRACTION: Iodine Status in Pregnant Women Having Urinary Fluoride in Contaminated Areas: A Case Study of Phayao Province

**DOI:** 10.1155/jeph/9761683

**Published:** 2026-04-01

**Authors:** Journal of Environmental and Public Health

RETRACTION: R. Somporn, C. Lapinee, C. Umponstira, R. Weterings, and S. Chaiwong, “Iodine Status in Pregnant Women Having Urinary Fluoride in Contaminated Areas: A Case Study of Phayao Province,” *Journal of Environmental and Public Health*, no. 2023 (2023), https://doi.org/10.1155/2023/3677359.

The above article, published online on 30 January 2023 in Wiley Online Library (https://wileyonlinelibrary.com), has been retracted by John Wiley & Sons Ltd.

The retraction has been agreed following an investigation of the concerns raised by a third party related to the scientifically incorrect description of hypothyroidism throughout the article. The investigation has identified that the article contains multiple incorrect claims concerning the role of TSH in the diagnosis of hypothyroidism, which in turn affects the integrity of the results presented in Tables 8 and 9.

In light of the above, the results can no longer be considered reliable, and it is therefore retracted.

The authors agree with this retraction.

